# Pronuclear morphology evaluation for fresh in vitro fertilization (IVF) and intracytoplasmic sperm injection (ICSI) cycles: a systematic review

**DOI:** 10.1186/1757-2215-6-64

**Published:** 2013-09-12

**Authors:** Alessia Nicoli, Stefano Palomba, Francesco Capodanno, Maria Fini, Angela Falbo, Giovanni Battista La Sala

**Affiliations:** 1 Department of Obstetrics, Gynecology and Pediatrics, A.O. Arcispedale S. Maria Nuova, IRCCS, University of Modena and Reggio Emilia, Viale Risorgimento 80, 42123 Reggio Emilia, Italy

**Keywords:** ARTs, Embryo, ICSI, IVF, Morphology, Zygote

## Abstract

The current systematic review was aimed to assess the effectiveness of the zygote morphology evaluation in fresh in vitro fertilization (IVF) and intracytoplasmic sperm injection (ICSI) cycles. All available studies reporting on zygote morphology and clinical and/or biological outcomes were analyzed. Forty studies were included in the final analysis. Fourteen different zygote scoring systems were employed. Zygote morphology correlated significantly with embryo quality and cleavage, blastocyst stage, embryonic chromosome status, in a high proportion of the studies which assessed the specific outcome [15/25 (60%), 15/20 (75%), 7/8 (87.5%), 6/6 (100%), respectively]. On the other hand, only a reduced proportion of papers showed a statistically significant relationship between implantation, pregnancy and delivery/live-birth rates and zygote morphology score [12/23 (52.2%), 12/25 (48%), 1/4 (25%), respectively]. In conclusion, our findings demonstrate the lack of conclusive data on the clinical efficacy of the zygote morphology evaluation in fresh IVF/ICSI cycles, even if biological results showing a good relationship with embryo viability suggest a role in cycles in which the transfer/freezing is performed at day 1.

## Introduction

The selection of the most competent embryos to transfer is a crucial point in in vitro fertilization (IVF) and intracytoplasmic sperm injection (ICSI) cycles in order to obtain the higher pregnancy rate reducing the risk of multiple pregnancy. To reach this aim a rapid, cheaper, standardized and non-invasive method of embryo classification would required.

Nowadays, only the study of the embryonic morphological features can concurrently satisfy all the above mentioned characteristics [1,2]. Over the years, various embryo scoring systems have been proposed [3]. They have been based on embryonic characteristic such as: number of blastomeres (commonly considered as the feature with the highest prognostic value), degree of fragmentation expressed as mild, moderate and severe (<10%, 10–25% and >25%, respectively), multinucleation of blastomeres (frequently associated with an higher abortion rate and number of chromosome abnormalities), and presence of vacuoles and/or aggregation of organelles, globally defined as cytoplasmic anomalies [4].

Despite the embryo morphology classification is a common practice in ARTs laboratories, its efficacy remains relatively low [5].

Additional information about the embryo competence could be obtained by the evaluation of the pronuclear (PN), nucleolar precursor bodies (NPBs) and polar bodies (PBs) alignment in the human zygote about 17 hours after the insemination [6,7]. Despite the correlation between the zygote morphology and the embryo competence have been studied by many Authors, the clinical efficacy of the zygote assessment is still debate [8,9].

Based on these considerations, we design the current study in order to review systematically the available scientific literature and to clarify the clinical efficacy of the zygote morphology assessment in fresh IVF and ICSI cycles.

## Materials and methods

### Information sources

We performed a systematic search using Medline and Web of Knowledge databases.

Keywords used for the search were: “human zygote” or “human pronuclear” and “morphology” or “evaluation” or “assessment”. Finally we performed a hand-search in the three main journals of reproductive medicine and biology, i.e. Fertility and Sterility, Human Reproduction and Human Reproduction Update.

We included in the search only full length papers in English language published between January 2000 and January 2013. Papers referenced in the articles found during the searches were also included in our analysis.

Studies conducted in animals or involving azoospermic patients and frozen oocytes, zygotes and/or embryos were excluded. Were also excluded duplicate reports, and papers obtained full text copies of all other papers.

### Definition of outcome measures

We selected all outcomes for which a potential correlation with zygote morphology had been hypothesized: embryo quality, cleavage and blastocyst stages, embryonic chromosome status, and implantation, pregnancy, and delivery/live-birth rates.

Our primary endpoint was to assess effect of zygote morphology on delivery/live-birth rate. All other clinical and/or biological outcome measures were considered secondary endpoints.

### Statistical analysis

To assess the correlation between zygote morphology, biological and clinical outcomes, Cox proportional-hazards model was used to calculate the odds ratio (OR) and its 95% confidence interval (CI) for each clinical endpoint.

Spearman’s rank correlations were used to test the influence of paper publication year on the correlation between the zygote morphology and ARTs outcomes.

The level of statistical significance was set at *P*<0.05 for all statistical analysis. The Statistics Package for Social Sciences (SPSS 14.0.1, 18 November 2005; SPSS Inc., Chicago, IL, US) was used for all calculations.

## Results

**Figure 1:**
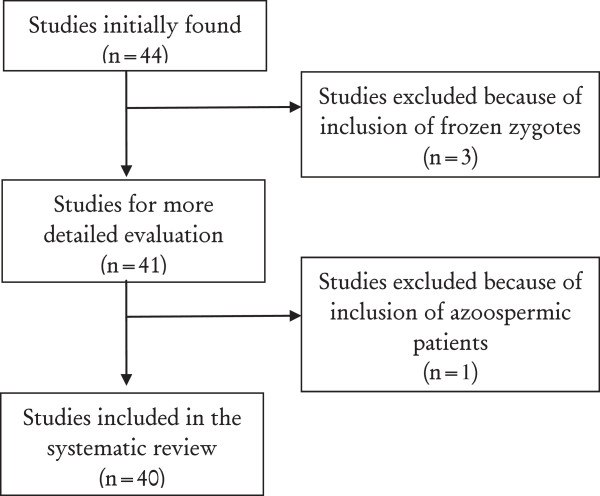
Study flow-chart.

In Figure 1 is shown the study flow-chart. Forty-four articles were initially found. After screening for inclusion and exclusion criteria, 40 papers were included in the current systematic review. Specifically, 3 studies were excluded because of the inclusion of frozen zygotes, oocytes or embryos; and 1 study because of the inclusion of azoospermic patients.

### Zygote morphology scoring systems used

A great heterogeneity was observed for the zygote morphology scoring systems used in the selected papers (Table 1). In fact, our systematic review identified a total of 15 zygote morphology scoring systems. Five scoring systems were original, whereas 10 derived from the previous (Table 1).

**Table 1:** Summary of the papers included in the analysis evaluating the relationship between zygote morphology and biological and clinical outcomes

**Authors**	**Design**	**Age (yrs)**	**Zygotes (n.)**	**ART procedure**	**TOPI**	**Zygote score**	**Outcome measures**						
							**EQ**	**CS**	**BS**	**IR**	**PR**	**D/LBR**	**ECS**
Scott et al. 2000 [10]	P	<43	3,701	IVF/ICSI	NA	Modified from Scott and Smith, 1998	Yes	NA	Yes	Yes	Yes	NA	NA
Wittemer et al. 2000 [11]	P	NA	1,000	IVF/ICSI	18h	Modified from Tesarik and Greco, 1999	Yes	NA	NA	NA	Yes	NA	NA
Tesarik et al. 2000 [12]	R	NA	350	ICSI	14–17h	Tesarik and Greco, 1999	Not	Not	NA	Yes	Yes	NA	NA
Ludwig et al. 2000 [13]	P	NA	405	IVF/ICSI	16–18h	Scott and Smith, 1998	Yes	NA	NA	NA	Yes	NA	NA
Balaban et al. 2001 [14]	R	NA	86	ICSI	14–17h	Tesarik and Greco, 1999	Yes	Yes	Yes	Yes	Yes	NA	NA
Salumets et al. 2001 [15]	P	NA	2,284	IVF/ICSI	16–18h	Scott and Smith, 1998/Tesarik and Greco, 1999	Not	Yes^*^	NA	Not	Not	NA	NA
Montag et al. 2001 [16]	P	NA	1,114	IVF/ICSI	16–20h	Modified from Tesaik and Greco, 1999	NA	NA	NA	Yes	Yes	NA	NA
De Placido et al. 2002 [17]	R	NA	1,171	ICSI	16–20h	Combination of Sadowy, 1998, Scott and Smith, 1998, Tesarik and Greco, 1999, Scott, 2000, and Wittemer, 2000	Not	Not	NA	Not	Not	NA	NA
Zollner et al. 2002 [18]	P	33.4±4.1	1,119	IVF/ICSI	16–18h	Number, juxtaposition and size of PN, number and alignment of NPBs	NA	Yes	Yes°°	NA	NA	NA	NA
Gianaroli et al. 2003 [19]	P	≥36	631	IVF/ICSI	16h	Gianaroli, 2003	Yes	Yes	NA	Yes	Yes	NA	Yes
Chen et al. 2003 [20]	P		368	IVF	18–21h	Modified from Scott, 2000	Not	NA	NA	NA	NA	NA	Yes
Nagy et al. 2003 [21]	P/R	25–40	912	ICSI	12–21h^§^	Size of PN, number and polarization of NPBs	Yes° (only in day 3)	Yes° (only in day 2)	NA	Yes^#^	Yes^#^	NA	NA
Scott et al. 2003 [22]	R	<43	3,882	IVF/ICSI	16–18h	Scott, 2000	NA	Yes	Yes	NA	NA	NA	NA
Lan et al. 2003 [23]	R	NA	1,894	IVF/ICSI	16–18h	Scott, 2000	Yes	Yes	Yes	Yes	Yes	NA	NA
Gámiz et al. 2003 [24]	P	NA	888	ICSI	16–18h	Size of PN, number, distribuiton and synchrony of NPBs	NA	Yes^***^	NA	NA	NA	NA	Yes^***^
Jaroudi et al. 2004 [25]	P	NA	131	IVF/ICSI	15–18h	Tesarik, 2000	Not	NA	NA	NA	Not	NA	NA
Kattera et al. 2004 [26]	P	35.4±3.6 (IVF) 35.1±3.4 (ICSI)	2,714	IVF/ICSI	18–20h	PN orientation	Yes	Yes	NA	Not	Not	NA	NA
Balaban 2004 [27]	R	NA	309	IVF/ICSI	17h	Modified from Tesarik and Greco, 1999	Yes	Yes	Yes	NA	NA	NA	Yes
Payne et al. 2005 [28]	P	≤42	552	IVF/ICSI	16–18h	Scott, 2000	Not	Not	NA	Not	Not	NA	NA
Edirisinghe et al. 2005 [29]	P	≥37	952	IVF/ICSI	16–18h	Scott, 2000	Yes	Yes	NA	NA	NA	NA	Yes
James et al. 2006 [30]	R	NA	3,333	IVF/ICSI	16–18h	Combination of Sadowy, 1998 and Scott, 2000	Not	NA	NA	Not	Not	Not	NA
Sjöblom et al. 2006 [31]	R	NA	1,961	IVF/ICSI	16–18h	PBs, NPBs and PN	NA	NA	Yes	NA	NA	NA	NA
Chen et al. 2006 [32]	P	35.7±3.7 (IVF) 35.5±3.4 (ICSI)	1,186	IVF/ICSI	18–20h	PN, PBs and NPBs	NA	Yes	NA	Not	Not	NA	NA
Gianaroli et al. 2007 [33]	R	38.2±3.7 (IVF) 39.0±3.5 (ICSI)	2,535	ICSI	16h	Gianaroli, 2003	Yes	NA	NA	Yes	NA	NA	Yes
Arroyo et al. 2007 [34]	P	33.1±2.93 (IVF) <39 (ICSI)	569	IVF/ICSI	14–23h	Tesarik and Greco, 1999 / Scott, 2000	Yes	NA	NA	Not	Not	NA	NA
Guerif et al. 2007 [35]	P	NA	4,042	IVF/ICSI	18–20h	Modified from Tesarik and Greco, 1999	NA	NA	Not	NA	NA	NA	NA
Scott et al. 2007 [36]	P	<38	2,528	IVF/ICSI	17–18h	Scott, 2000	NA	NA	NA	Yes	Yes	Yes	NA
Depa–Martynow et al. 2007 [37]	P	NA	787	IVF	16–18h	Scott, 2000	Yes	NA	NA	NA	NA	NA	NA
Nicoli et al. 2007 [38]	R	23–41	1,032	IVF/ICSI	18–20h	Modified from Scott, 2000 and Gianaroli, 2003	Not	NA	NA	Not	Not	NA	NA
Álvarez et al. 2008 [39]	R	35.0±3.5	883	IVF/ICSI	16–18h	Tesarik and Greco, 1999	Yes	NA	NA	Yes	Yes	NA	NA
Liu et al. 2008 [40]	P	30.4±3.71 (IVF) 30.1±3.79 (ICSI)	2,836	IVF/ICSI	16–20h	Scott, 2000	Yes	Yes	NA	Yes	Not	NA	NA
Qian et al. 2008 [41]	P	NA	973	IVF/ICSI	18–18h	Scott, 2000/Lan, 2003	Not	NA	NA	NA	NA	NA	NA
Brezinova et al. 2009 [42]	R	<39	1,954	IVF/ICSI	16–20h	Modified from Tesarik and Greco, 1999	NA	NA	NA	Not	Not	NA	NA
Maille et al. 2009 [43]	P	40.1±1.3 (IVF) 27.1±1.4 (ICSI)	301	ICSI	16–18h	Gianaroli, 2003	Not	Yes ^**^	NA	Yes	Yes ^**^	NA	NA
Zamora et al. 2010 [44]	P	NA	2,105	IVF/ICSI	16–18h	PN size, NPBs and PBs	NA	Yes^ç^	NA	NA	NA	NA	NA
Weitzman et al. 2010 [45]	P	<36	852	IVF/ICSI	18–20h	Tesarik and Greco/Scott, 2000	NA	NA	NA	Not	NA	NA	NA
Nicoli et al. 2010 [46]	R	35.9±4.0	1,078	IVF/ICSI	18–20h	Gianaroli, 2003	Not	Not	NA	NA	Not	Not	NA
Bar–Yoseph et al. 2011 [47]	R	31.1±5.0	1,516	IVF/ICSI	17–18h	Scott, 2000	NA	NA	NA	Not	NA	NA	NA
Aydin et al. 2011 [48]	P	29.8±3.5	487	ICSI	16–18h	Modifed from Tesarik and Greco, 1999/Scott, 2000	NA	Not	NA	NA	NA	NA	NA
Nicoli et al. 2013 [49]	R	36.6±3.9	755	IVF/ICSI	18–20h	Gianaroli, 2003	NA	NA	NA	NA	Not	Not	NA

*P*: prospective; *R*: retrospective; *IVF*: in vitro fertilization; *ICSI*: intracytoplasmic spem injection; *PN*: pronuclei; *PBs*: polar bodies; *NPBs*: nucleolar precursor bodies; *TOPI*: time of observation post–insemination; *Yes*: statistically significant correlation; *Not*: No correlation or correlation not statistically significant; *NA*: not available.

*: only for zygotes classified according to Scott and Smith, 1998; °°: only in ICSI cycles; °: only for polarization, and not for number of NPBs ^§^: first observation 12–14 h post–ICSI, second observation 16–18 h post ICSI (retrospective) and third observation ≥ 20 h post ICSI (prospective); #: only in combination with embryo morphology evaluation; ***: only in patients < 37 years old; **: only in patients < 30 years old; ç: only for NPBs.

Below we will describe firstly the original and modified scores, and that proposed in a recent European Society of Human Reproduction and Embryology (ESHRE) consensus workshop on embryo assessment, and secondly we will analyze the biological and clinical outcomes.

### Original scores

**Figure 2:**
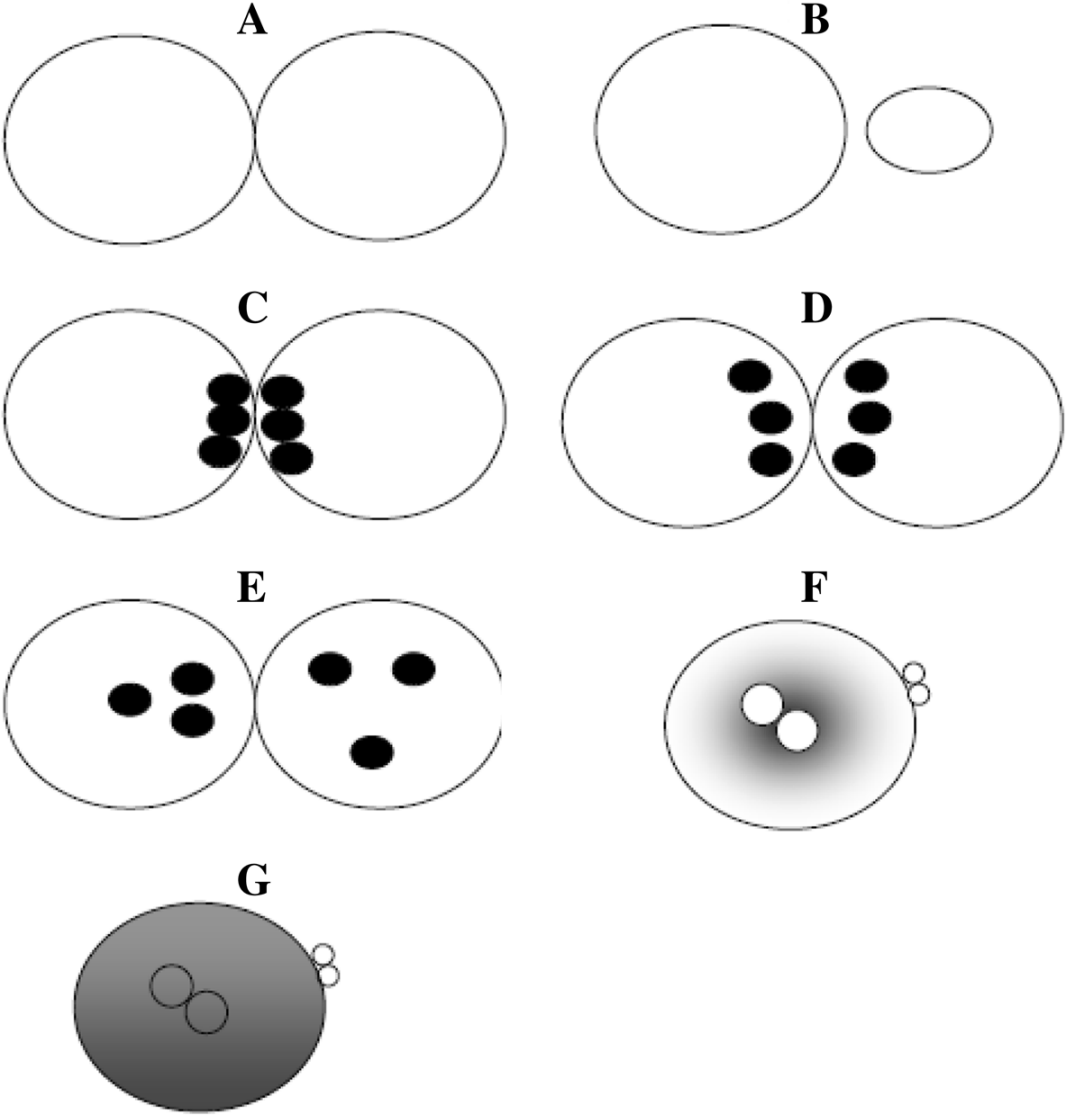
**Zygote scoring system of Scott and Smith **[50]**.** In this classification, if PN are close or aligned they are assigned a score 5 **(A).** If they are well separated or unequal in size they are classified as score 1 **(B)**. Nucleoli aligned in a row at the PN junction are scored as 5 **(C)**, beginning to align as 4 **(D)**, scattered as 3 **(E)**. The cytoplasm is scored as follow: heterogeneous in appearance with a clear halo around the edges, occasionally with a clear area in the centre around the PN and darkened ring/halo in the middle, scored 5 **(F)**, whereas zygotes with a clear homogeneous cytoplasm or a pitted and/or darkened cytoplasm scored 6 **(G)**.

In 1998, Scott and Smith was the first to develop a zygote scoring system [50]. In this classification, if PN are close or aligned they are assigned a sore 5 (Figure 2A). If they are well separated or unequal in size they are classified as score 1 (Figure 2B). NPBs aligned in a row at the PN junction are scored as 5 (Figure 2C), beginning to align as 4 (Figure 2D), scattered as 3 (Figure 2E). The cytoplasm is scored as follow: heterogeneous in appearance with a clear halo around the edges, occasionally with a clear area in the centre around the PN and darkened ring/halo in the middle, score 5 (Figure 2F). Zygotes with a clear homogeneous cytoplasm or a pitted and/or darkened cytoplasm scored 6 (Figure 2G) [50].

**Figure 3:**
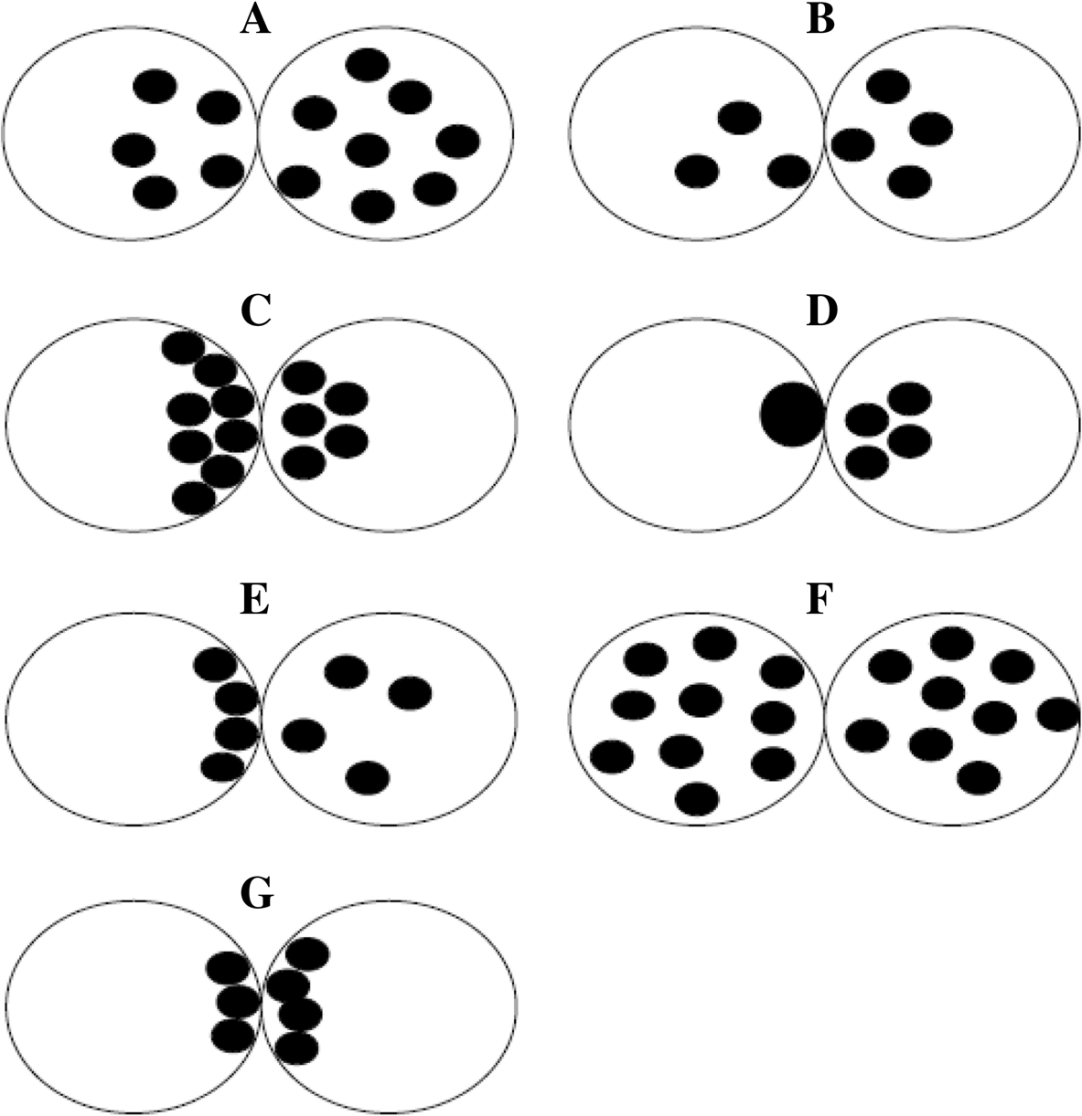
**Zygote scoring system of Tesarik and Greco **[51]**.** pattern 1 includes zygotes with big difference (>3) in the number of NPBs in both PN **(A)**, pattern 2 includes zygotes showing a small number (<7) of NPBs without polarization in at least one PN **(B)**, pattern 3 includes zygotes with a large number (>7) of NPBs with polarization in at least one PN **(C)**, pattern 4 includes zygotes characterized by a very small number (<3) of NPBs in at least one PN **(D)** and pattern 5 includes zygotes showing polarized distribution of NPBs in one PN and non-polarized in the other. These 5 patterns are considered as “abnormal”; zygotes not included in pattern 1–5 are classified as pattern 0, and are considered “normal” **(F** and **G)**.

The scoring system introduced by Tesarik and Greco in 1999 included 6 different patterns based on NPBs features [51]. Specifically, pattern 1 includes zygotes with big difference (>3) in the number of NPBs in both PN (Figure 3A), pattern 2 includes zygotes showing a small number (<7) of NPBs without polarization in at least one PN (Figure 3B), pattern 3 includes zygotes with a large number (>7) of NPBs with polarization in at least one PN (Figure 3C), pattern 4 includes zygotes characterized by a very small number (<3) of NPBs in at least one PN (Figure 3D) and pattern 5 includes zygotes showing polarized distribution of NPBs in one PN and non-polarized in the other. These 5 patterns are considered as “abnormal”; zygotes not included in pattern 1–5 are classified as pattern 0, and are considered “normal” (Figure 3F and G) [51].

**Figure 4:**
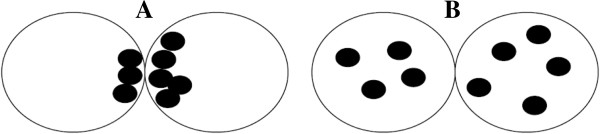
**Zygote scoring system of Tesarik et al. **[12]**.** Zygotes showing a normal morphology are classified as “pattern 0”, and zygotes showing an abnormal morphology are classified as “non-pattern 0”. Specifically, pattern 0 include zygotes with a difference in a NPBs number between the two PN < 3, the same distribution (random or polarized) of NPBs in both PN, and at least one NPB in each PN **(A** and **B)**. All the other NPBs configurations lead to classified zygotes as “non-pattern 0”.

Tesarik et al. [12] proposed another zygote morphology classification in 2000. In this system, zygotes showing a normal morphology are classified as “pattern 0”, and zygotes showing an abnormal morphology are classified as “non-pattern 0”. Specifically, pattern 0 include zygotes with a difference in a NPBs number between the two PN < 3, the same distribution (random or polarized) of NPBs in both PN, and at least one NPB in each PN (Figure 4A and B). All the other NPBs configurations lead to classified zygotes as “non-pattern 0” [12].

**Figure 5:**
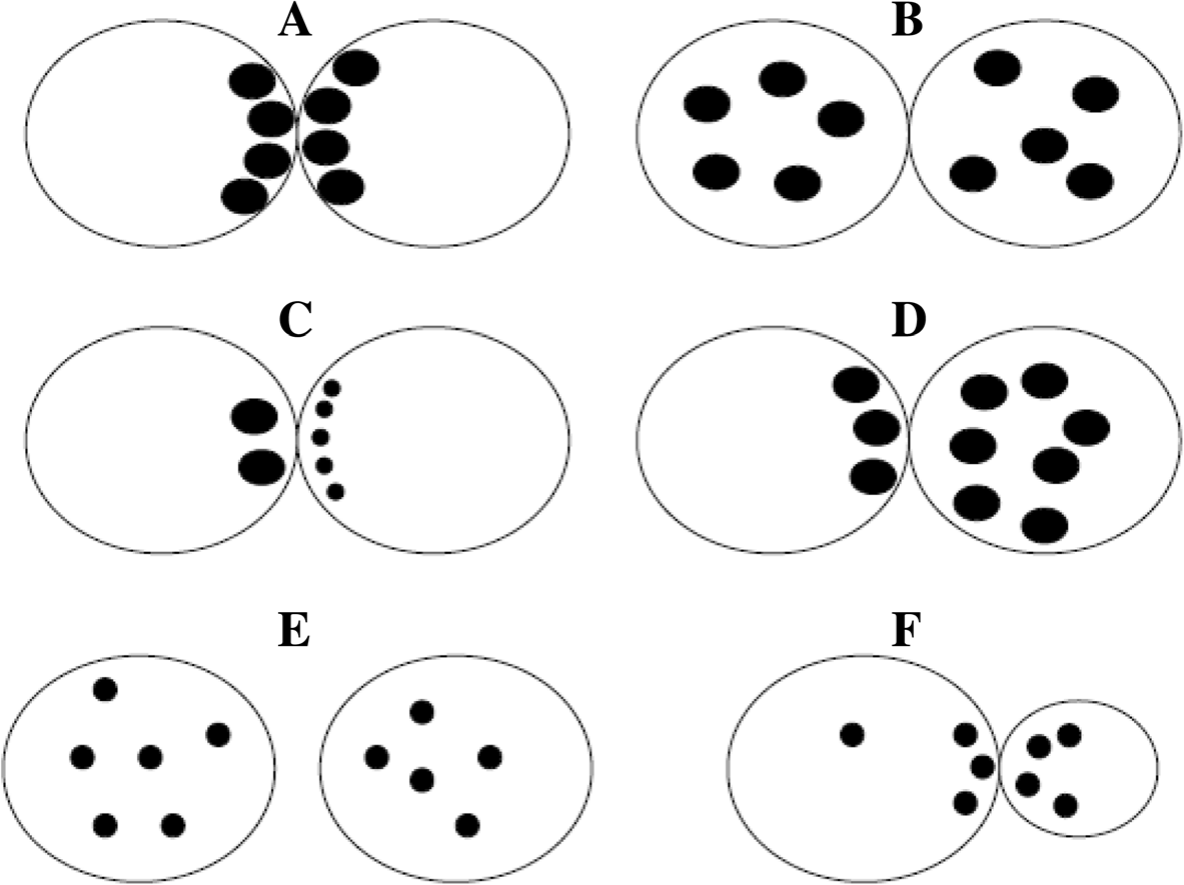
**Zygote scoring system of Scott et al. **[10]**.** Z1 includes zygotes with equal number of nucleoli aligned at PN junction **(A)**, Z2 includes zygotes with equal number and size of nucleoli (between 3 and 7) which are equally scattered in the two PN **(B)**, Z3 includes zygotes with either very small/large nucleoli **(C** and **D)**, and Z4 includes zygotes showing PN separated or different in size and small nucleoli, partially aligned or scattered **(E** and **F)**.

In the same year, Scott et al. [10] introduced a new classification for zygote morphology scoring including 4 patterns (Z1-Z4). In particular, Z1 includes zygotes with equal number of NPBs aligned at PN junction (Figure 5A), Z2 includes zygotes with equal number and size of NPBs (between 3 and 7) which are equally scattered in the two PN (Figure 5B), Z3 includes zygotes with either very small/large NPBs (Figure 5C and D), and Z4 includes zygotes showing PN separated or different in size and small NPBs, partially aligned or scattered (Figure 5E and F) [10].

**Figure 6:**
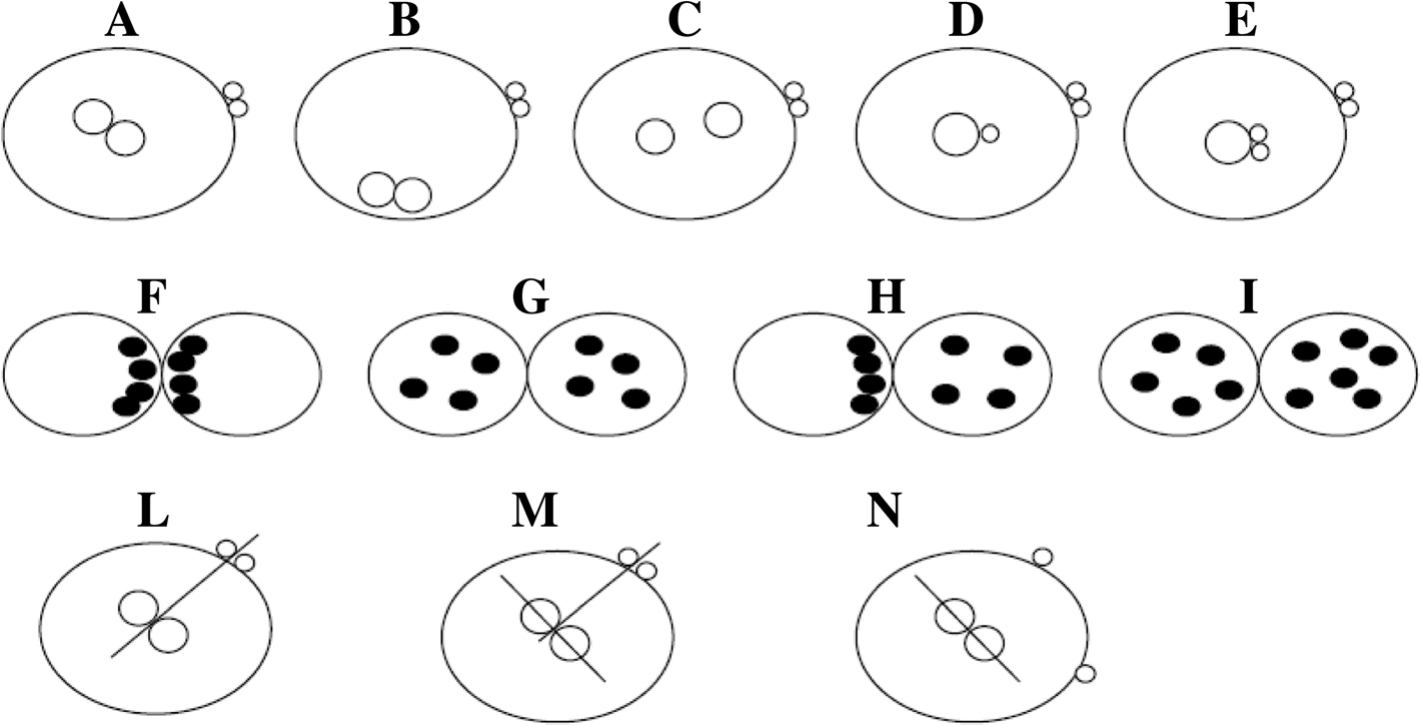
**Zygote scoring system of Gianaroli et al. **[19]**.** They identified 5 different patterns according to PN morphology: (i) juxtaposed and centralized **(A)**, (ii) juxtaposed and peripheral **(B)**, (iii) centralized and separated **(C)**, (iv) unequal size **(D)**, and (v) fragmented **(E)**. At regard to nucleolar morphology, 4 different patterns were proposed: (i) large size, aligned **(F)**, (ii) large sized scattered in both PN **(G)**, (iii) large size, aligned in one PN and scattered in the other **(H)**, (iv) small size in at least one PN, scattered **(I)**. Finally, the orientation of polar bodies was described in relation to the longitudinal axis of PN: (i) the longitudinal axis **(L)**; (ii) perpendicular to the longitudinal axis **(M)**; (iii) in different position **(N)**.

The scoring system used by Gianaroli et al. [19] in 2003 included the evaluation of PN, NPBs and the orientation of PBs. The Authors identified 5 different patterns according to PN morphology: (i) juxtaposed and centralized (Figure 6A), (ii) juxtaposed and peripheral (Figure 6B), (iii) centralized and separated (Figure 6C), (iv) unequal size (Figure 6D), and (v) fragmented (Figure 6E). At regard to NPBs morphology, 4 different patterns were proposed: (i) large size, aligned (Figure 6F), (ii) large sized scattered in both PN (Figure 6G), (iii) large size, aligned in one PN and scattered in the other (Figure 6H), (iv) small size in at least one PN, scattered (Figure 6I). Finally, the orientation of PBs was described in relation to the longitudinal axis of PN: (i) the longitudinal axis (Figure 6L); (ii) perpendicular to the longitudinal axis (Figure 6M); (iii) in different position (Figure 6N).

### Modified scores

#### Modified Tesarik and Greco scoring systems

Six zygotes morphology scoring systems derived from the zygote scoring proposed by Tearik and Greco [51] are available in literature.

**Figure 7:**
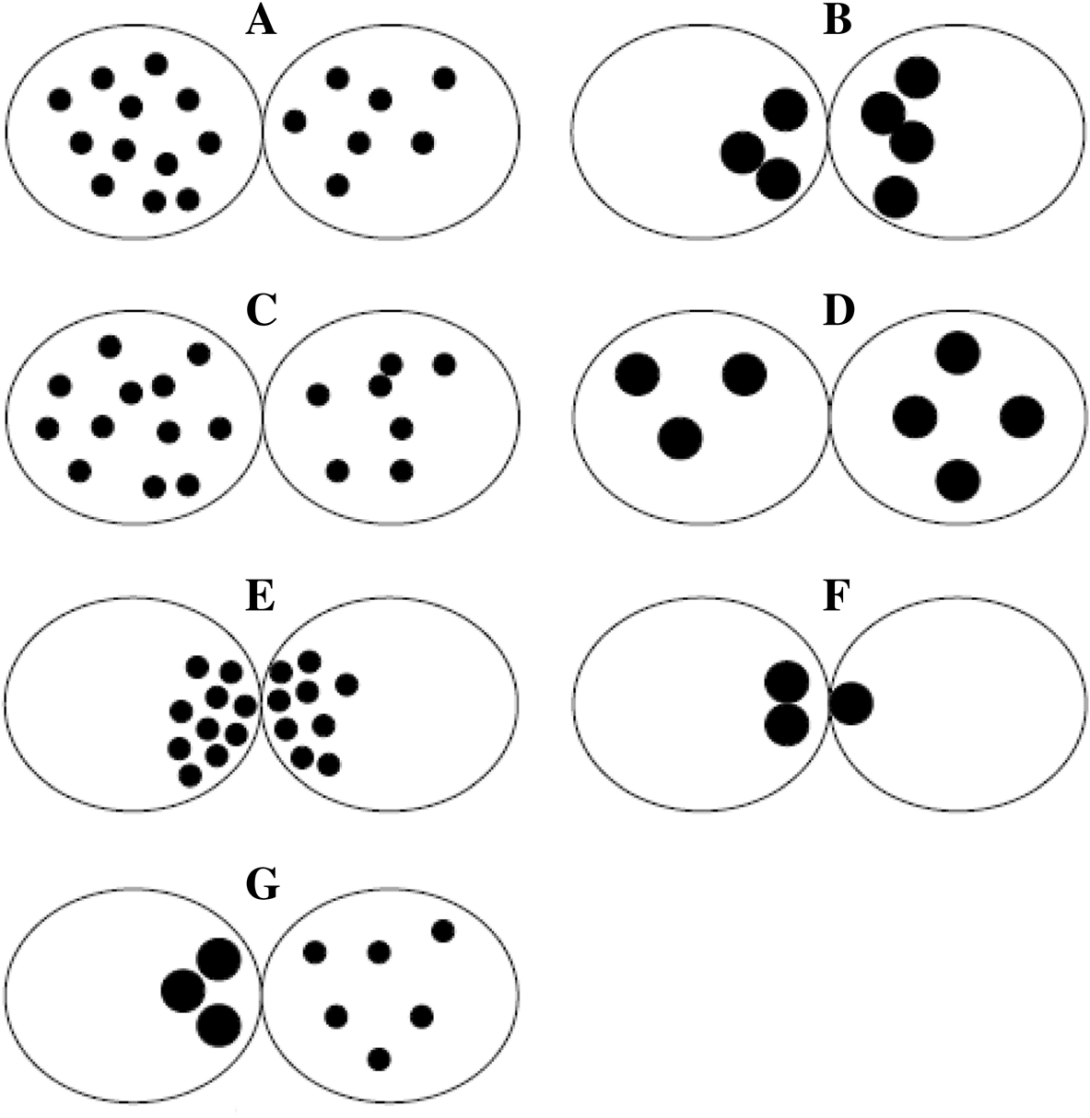
**Zygote scoring system of Wittemer et al. **[11]**.** They defined zygotes with “normal” pattern 0 as zygotes with the four following characteristics: (i) the number of NPBs never differed by more than 3 **(A)**; (ii) NBPs always polarized when fewer than 7 and never polarized if more than 7 in at least one PN **(B** and **C)**; (iii) the number of NBPs in PN never fewer than 3 **(D)**; (iv) the distribution of NPBs either polarized or not in both PN **(E** and **F)**. Whereas zygotes that did not conform to this morphological pattern were considered as “abnormal”.

In 2000, Wittemer et al. [11] defined zygotes with “normal” pattern 0 as zygotes with the four following characteristics: (i) the number of NPBs never differed by more than 3 (Figure 7A); (ii) NBPs always polarized when fewer than 7 and never polarized if more than 7 in at least one PN (Figure 7B and C); (iii) the number of NBPs in PN never fewer than 3 (Figure 7D); (iv) the distribution of NPBs either polarized or not in both PN (Figure 7E and F). Whereas zygotes that did not conform to this morphological pattern were considered as “abnormal” [11].

**Figure 8:**
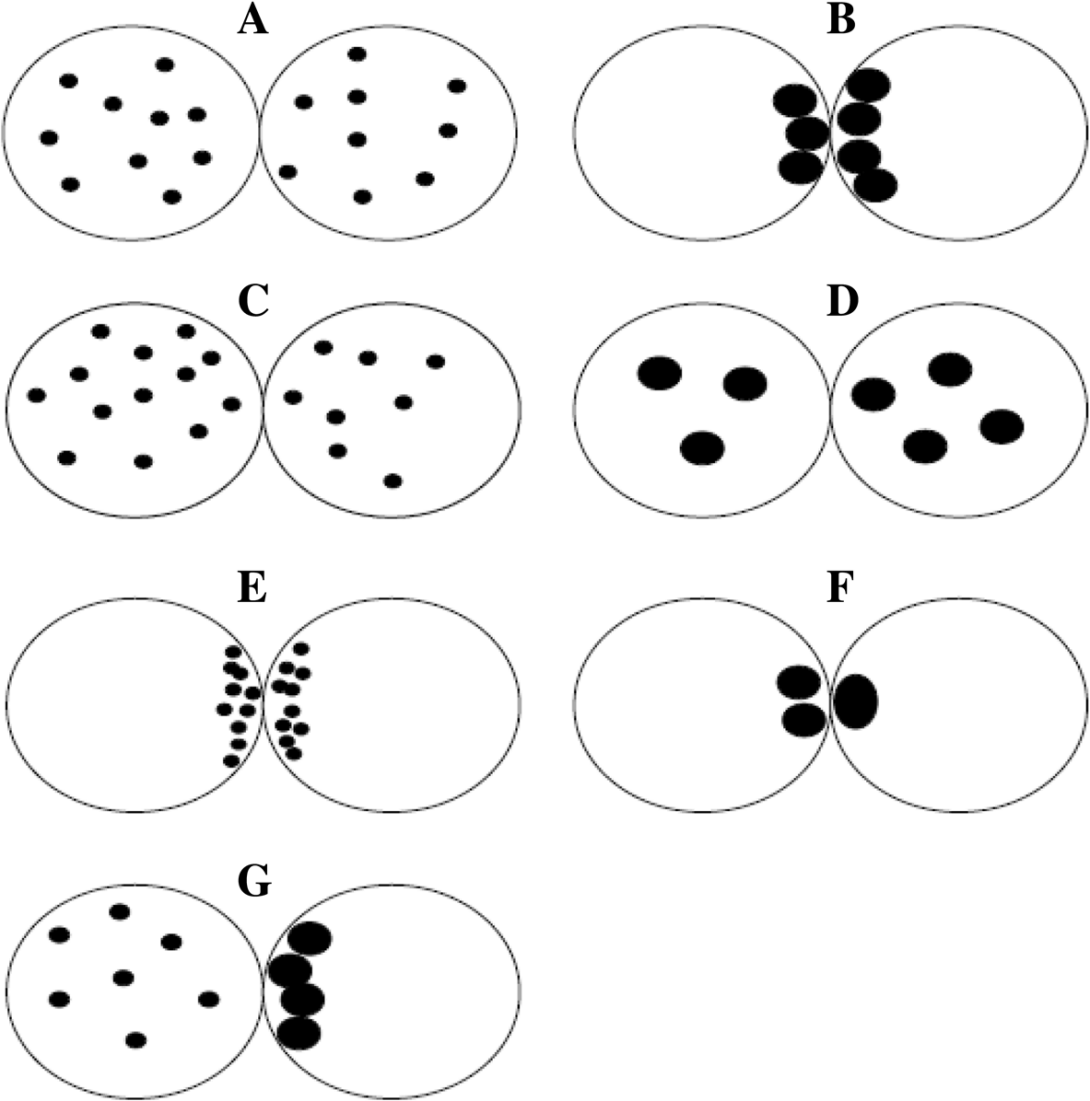
**Zygote scoring system of Montag et al. **[16]**.** This classification subdivided the zygotes with a “normal” pattern 0 in two further different patterns according to NPBs number and distribution: pattern 0A **(A)** (>7 equally distributed NPBs) and pattern 0B (≤ polarized NPBs) **(B)**.

One year later, Montag et al. [16] subdivided the zygotes with a “normal” pattern 0 in two further different patterns according to NPBs number and distribution: pattern 0A (Figure 8A) (>7 equally distributed NPBs) and pattern 0B (≤ polarized NPBs) (Figure 8B) [16].

**Figure 9:**
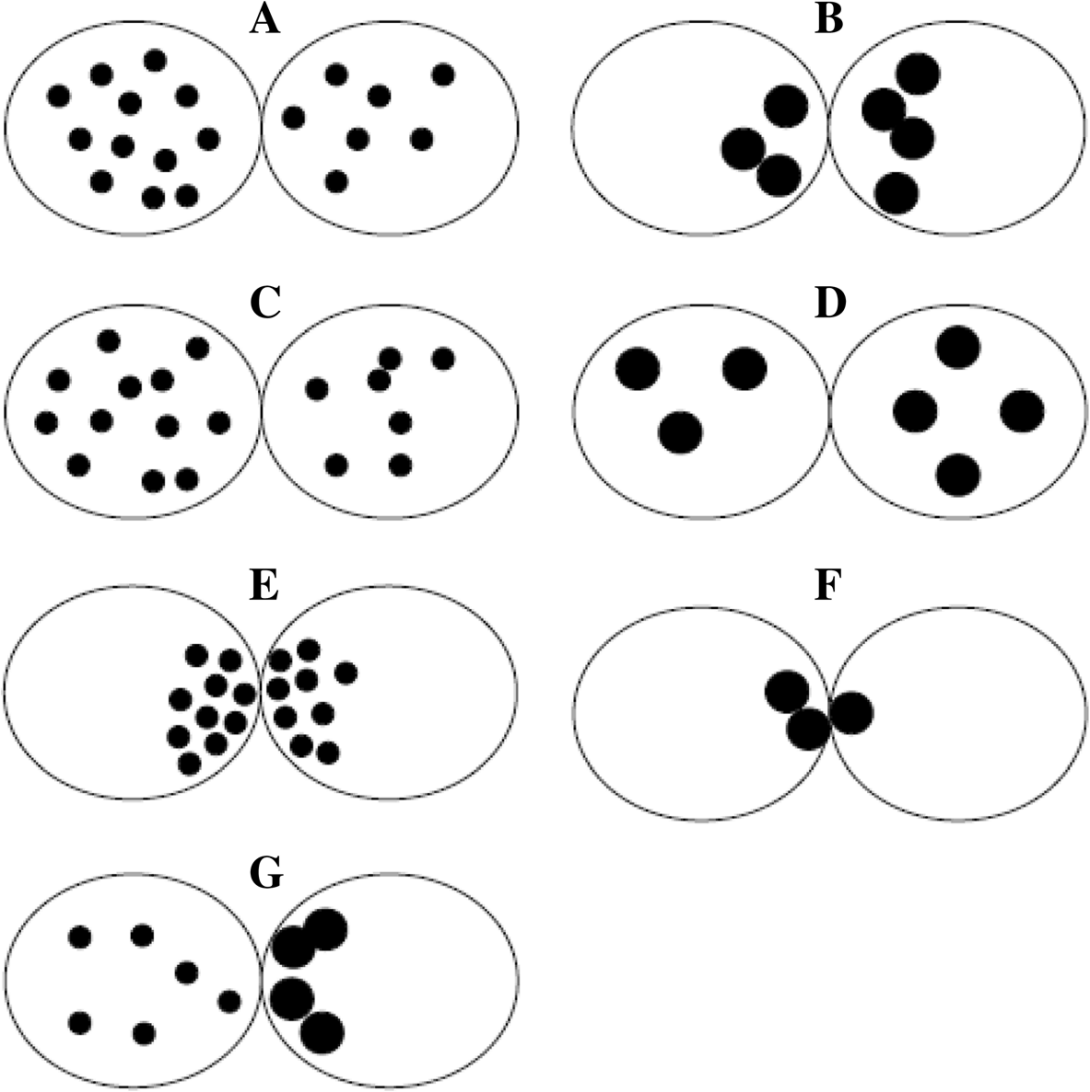
**Zygote scoring system of Balaban et al. **[27]**.** The authors identified two group: group 1, zygotes corresponding to “pattern 0” of the original classification **(A** and **B)**; group 2, zygotes showing a single PN; group 3, zygotes with 2PN originally classified as pattern 1–5 **(C**-**G)**.

In 2004, Balaban et al. [27] classified the zygotes in 3 different groups: group 1, zygotes corresponding to “pattern 0” of the original classification (Figure 9A and B); group 2, zygotes showing a single PN; group 3, zygotes with 2PN originally classified as pattern 1–5 (Figure 9C-G) [27].

**Figure 10:**
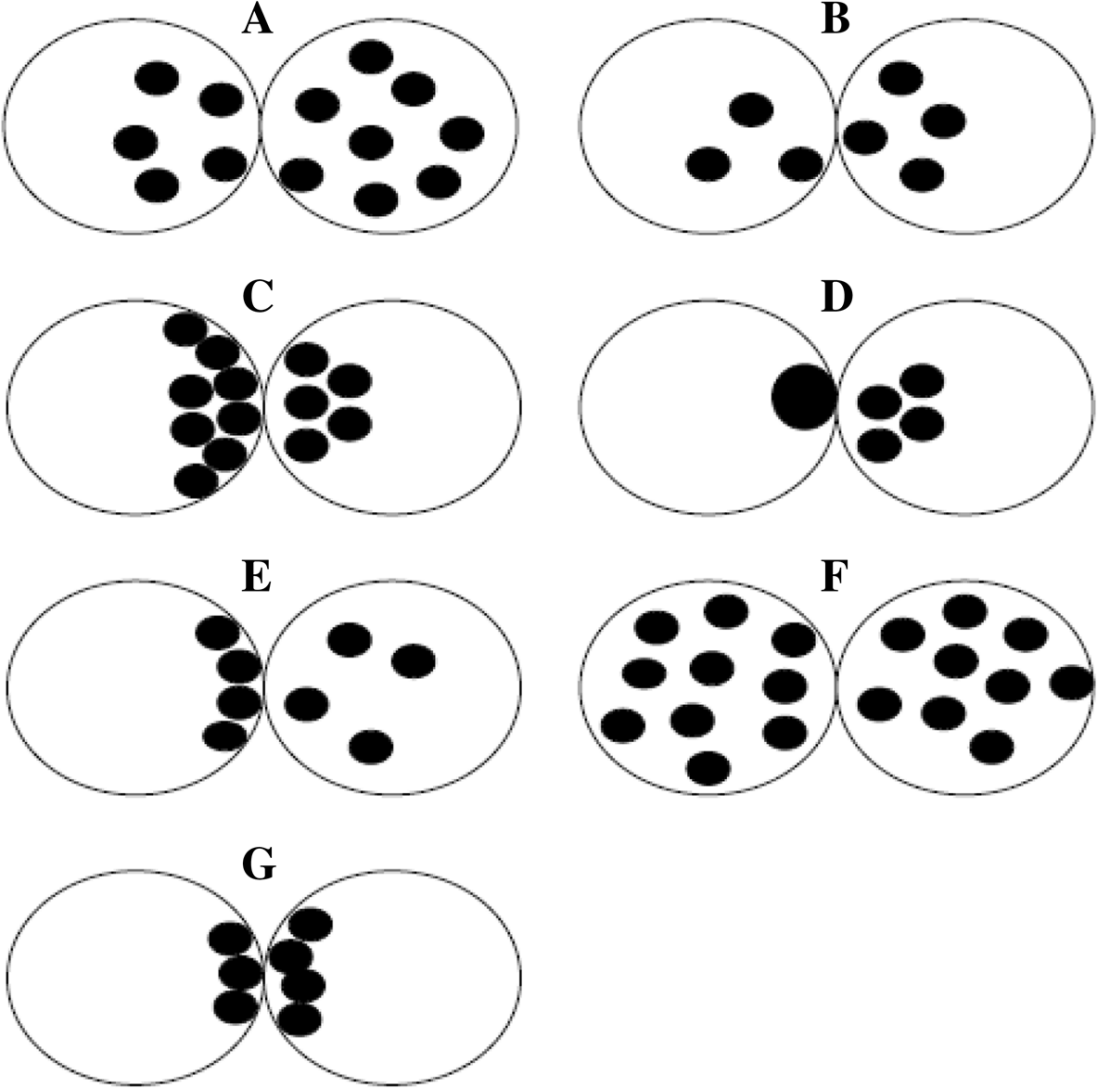
**Zygote scoring system of Guerif et al. **[35]**.** This classification grouped the patterns 1–5 as defined by Tesarik and Greco [51] into a single class called “non-pattern 0” **(A**-**E)**. Zygotes with “non-pattern 0” were considered abnormal zygotes in opposition to “pattern 0” zygotes (normal zygotes) **(F** and **G)**.

In 2007, Guerif et al. [35] simplified the criteria initially described by Tesarik and Greco grouping the patterns 1–5 into a single class called “non-pattern 0” (Figure 10A-E). Zygotes with “non-pattern 0” were considered abnormal zygotes in opposition to “pattern 0” zygotes (normal zygotes) (Figure 9F and G) [35].

**Figure 11:**
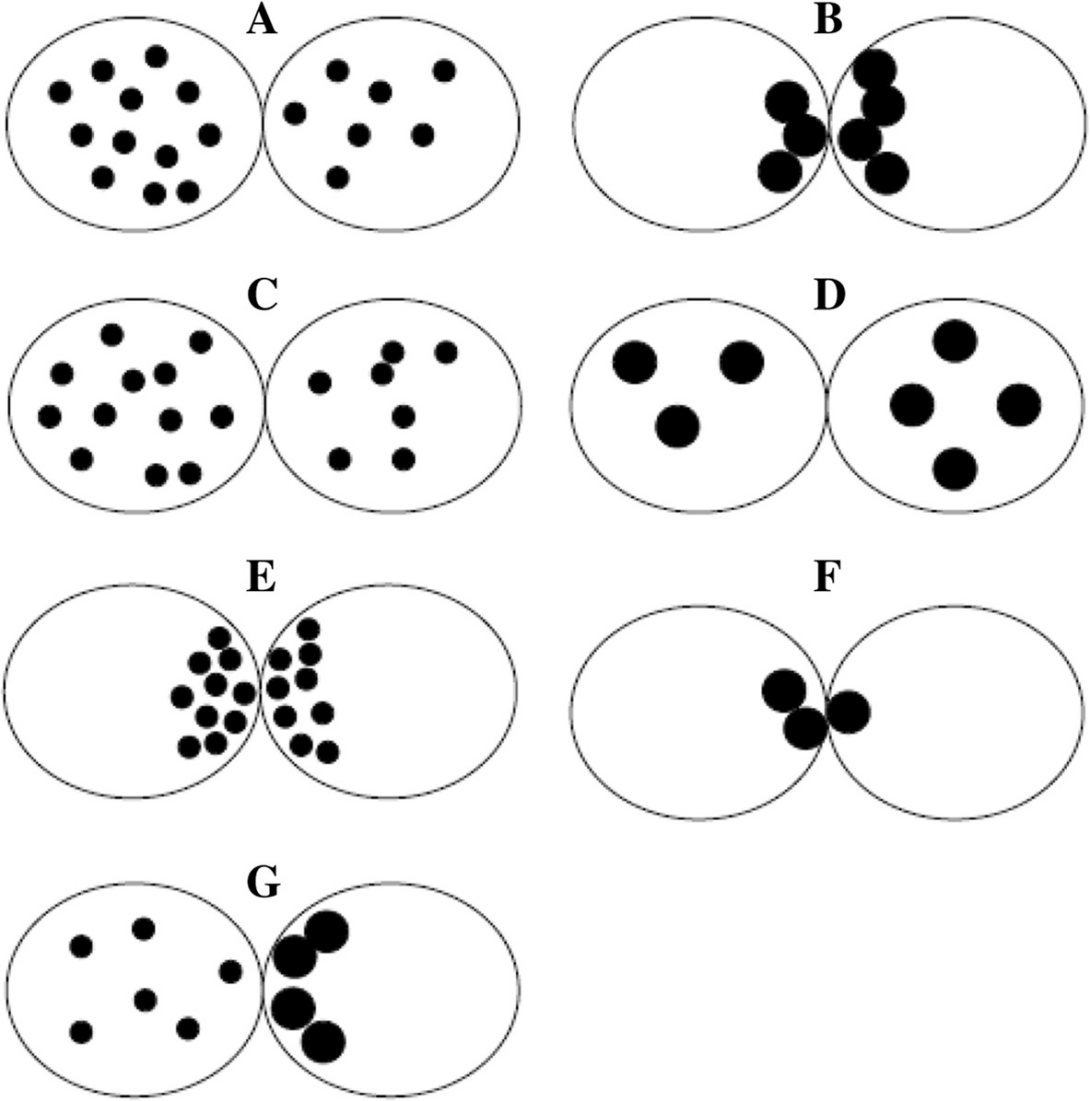
**Zygote scoring system of Brezinova et al. **[42]**.** Zygotes exhibiting some number of NPBs evenly distributed in the PN or large NBPs with polarized distribution between the two PN were grouped in pattern 0 **(**and **B)**, whereas all the other non symmetrical alignments of NPBs were classified as pattern “other” **(C**-**G)**.

In 2009, Brezinova et al. [42] also published a simplification of the original zygotes scoring system. Specifically, zygotes exhibiting some number of NPBs evenly distributed in the PN or large NPBs with polarized distribution between the two PN were grouped in pattern 0 (Figure 11A and B), whereas all the other non symmetrical alignments of NPBs were classified as pattern “other” (Figure 11C-G) [42].

**Figure 12:**
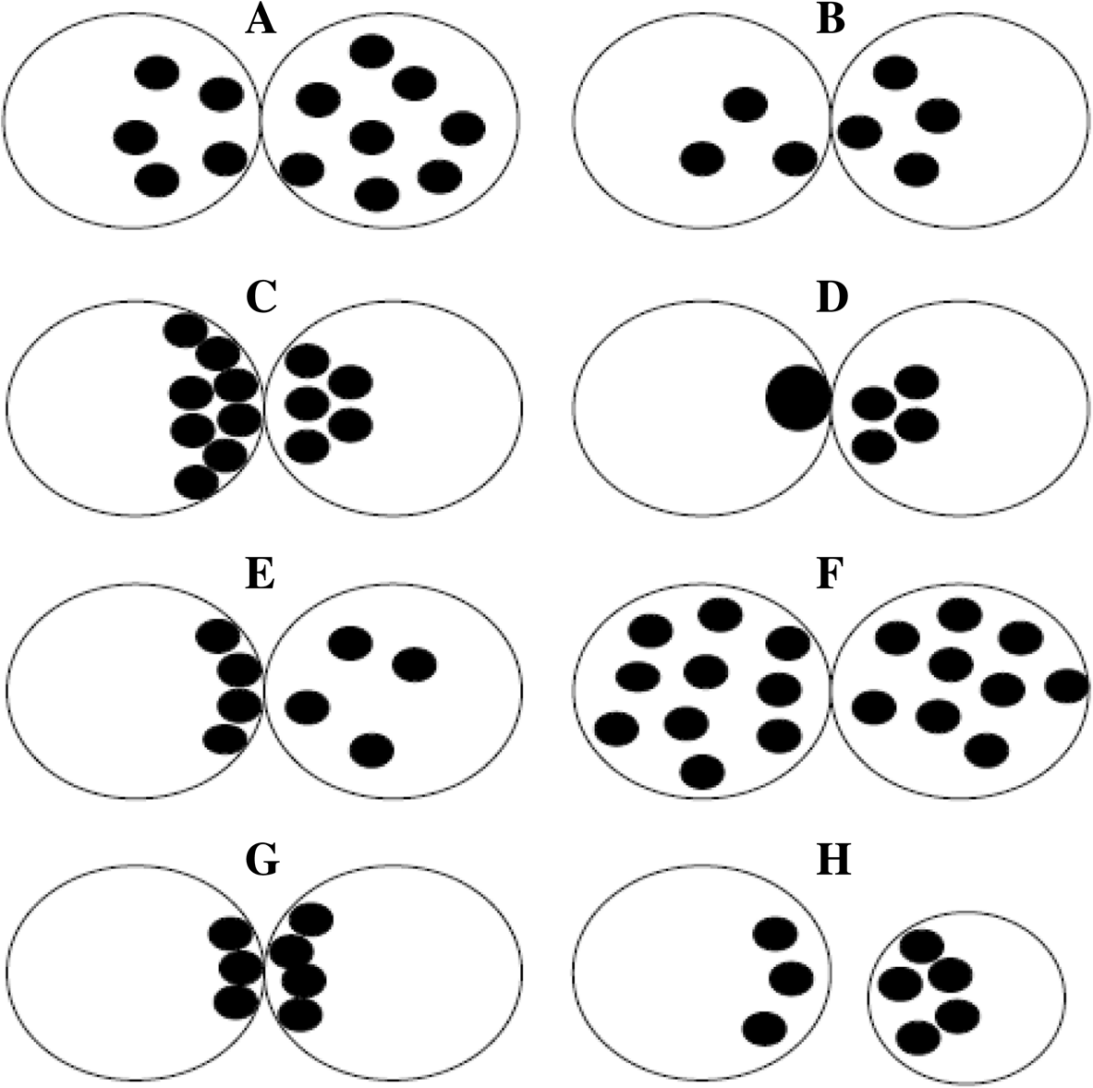
**Zygote scoring system of Aydin et al. **[48]**.** In the zygote scoring system another pattern was adding to those originally described [51]**(A**-**G)**. Zygotes presenting disconnected PN with unequal size and difference in the number of NPBs less than 3 in both PN were included in this new pattern **(H)**.

Finally, in the zygote scoring system adopted by Aydin et al. [48] another pattern was adding to those originally described [51] (Figure 12A-G). Zygotes presenting disconnected PN with unequal size and difference in the number of NPBs less than 3 in both PN were included in this new pattern (Figure 12H) [48].

#### Modified Scott scoring systems

In one paper [23] was used a scoring system derived from that initially described by Scott et al. [10].

**Figure 13:**
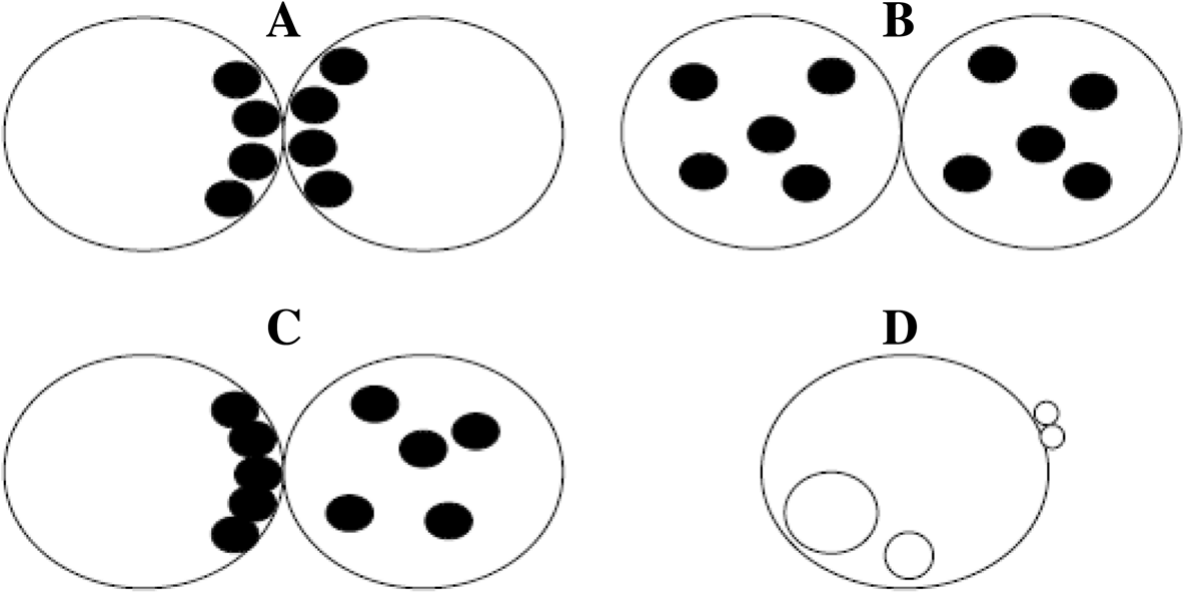
**Zygote scoring system of Lan et al. **[23]**.** Zygote Z1 had equal number of nucleoli (between 3 and 7) aligned at the PN junction **(A)**, Z2 had nucleoli equally in number and size equally scattered in both PN **(B)**, Z3 had equal number of nucleoli of equal size in the same PN but with one PN having alignment at the PN junction and the other with scattered nucleoli **(C)**, and Z4 had PN not aligned, grossly different in size or not located in the central part of the zygote **(D)**. Zygotes with unequal number (a difference of more than one nucleolus), and/or size of nucleoli were considered as Z3.

In 2003, Lan et al. [23] included further characteristics to classify zygote morphology in 4 patterns. Briefly, Z1 had equal number of NPBs (between 3 and 7) aligned at the PN junction (Figure 13A), Z2 had NPBs equally in number and size equally scattered in both PN (Figure 13B), Z3 had equal number of NPBs of equal size in the same PN but with one PN having alignment at the PN junction and the other with scattered NPBs (Figure 13C), and Z4 had PN not aligned, grossly different in size or not located in the central part of the zygote (Figure 13D). Zygotes with unequal number (a difference of more than one nucleolus), and/or size of NPBs were considered as Z3 [23].

#### Modified scoring systems derived from combination of multiple original scoring systems

In three included papers a zygote scoring system was developed combining multiple previous classifications [17,38].

**Figure 14:**
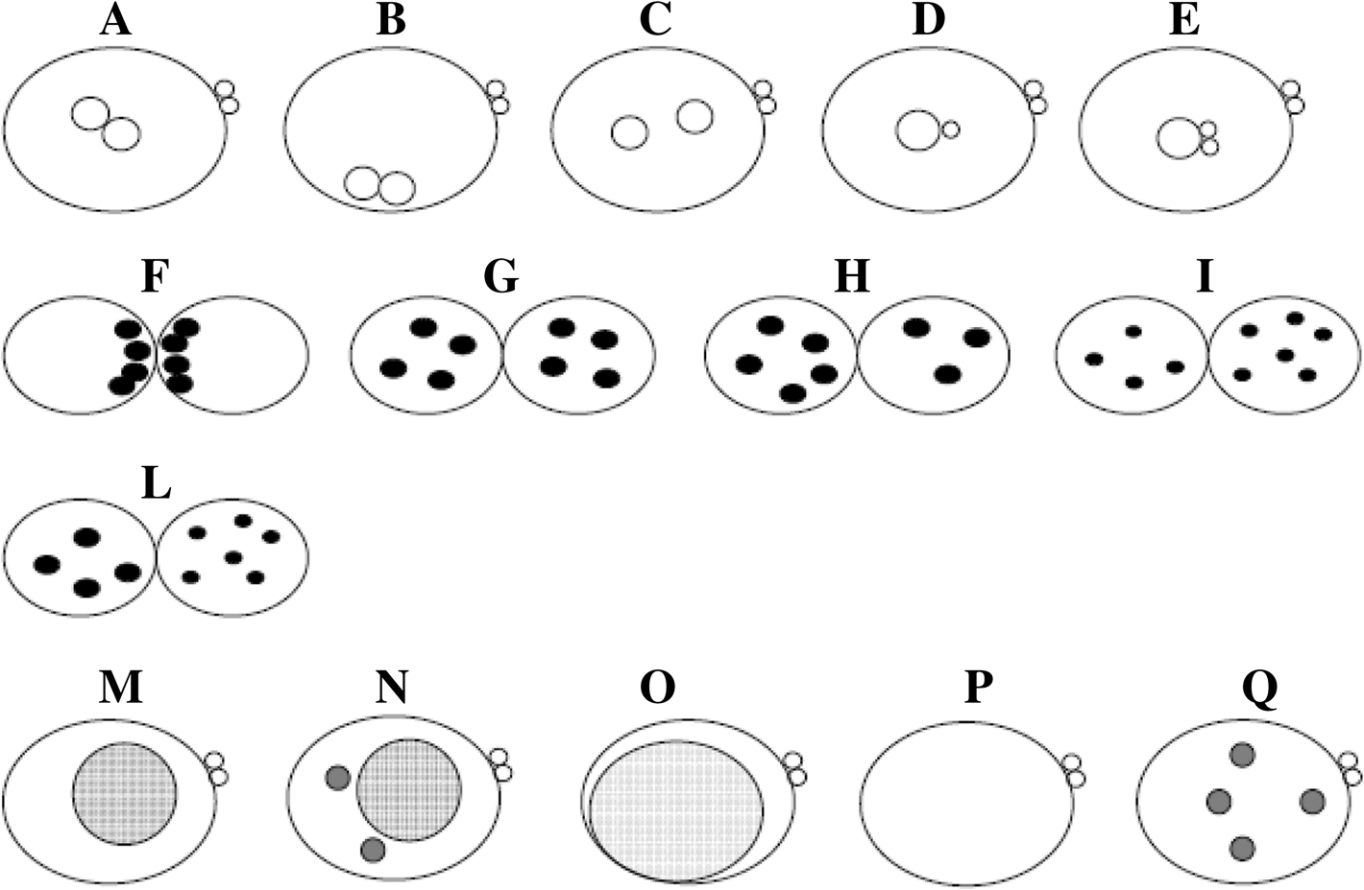
**Zygote scoring system of De Placido et al. **[17]**.** The authors combined five previous scoring systems [10,11,50-52] and considered 3 main parameters: (i) the position of PN in relation to the cytoplasm **(A**-**E)**; (ii) the morphology and orientation of nucleoli **(F**-**L)**; (iii) the presence of a dense area of the cytoplasm aggregate around the PN (cytoplasmic flare) **(M**-**Q)**. Zygotes showing two opposed PN with equal size in centre of the cytoplasm, equal number of juxtaposed nucleoli and the cytoplasmic flare were considered as top quality zygotes.

In 2002, De Placido et al. [17] combined five previous scoring systems [10,11,50-52] and considered 3 main parameters: (i) the position of PN in relation to the cytoplasm (Figure 14A-E); (ii) the morphology and orientation of NPBs (Figure 14F-L); (iii) the presence of a dense area of the cytoplasm aggregate around the PN (cytoplasmic flare) (Figure 14M-Q). Zygotes showing two opposed PN with equal size in centre of the cytoplasm, equal number of juxtaposed NPBs and the cytoplasmic flare were considered as top quality zygotes [17].

**Figure 15:**
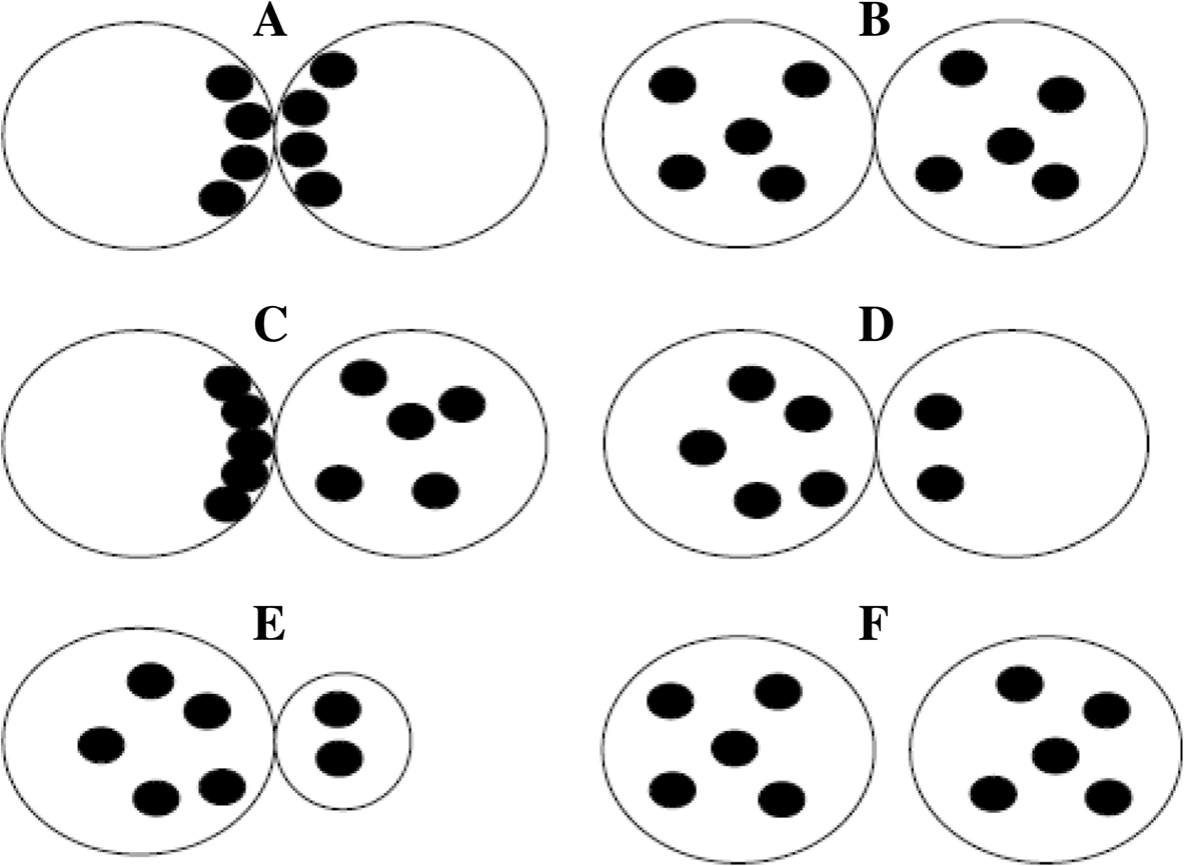
**Zygote scoring system of James et al. **[30]**.** Zygotes were grounded in scores from 1 to 4: score 1, zygotes with equal numbers of nucleoli that are aligned at the furrow between the PN **(A)**; score 2, zygotes with equal numbers of nucleoli that are not aligned at the furrow **(B)**; score 3, zygotes with marked differences in size and/or number of nucleoli with nucleoli not aligned **(C** and **D)**; and score 4, zygotes with different size PN, non central PN or PN that were not in contact with each other **(E** and **F)**.

In 2006, James et al. [30] described a zygote scoring system using the previous pronuclear scores by Sadowy et al. [52] and Scott et al. [10]. In particular, zygotes were grounded in scores from 1 to 4: score 1, zygotes with equal numbers of NPBs that are aligned at the furrow between the PN (Figure 15A); score 2, zygotes with equal numbers of NPBs that are not aligned at the furrow (Figure 15B); score 3, zygotes with marked differences in size and/or number of NPBs with NPBs not aligned (Figure 15C and D); and score 4, zygotes with different size PN, non central PN or PN that were not in contact with each other (Figure 15E and F) [30].

**Figure 16:**
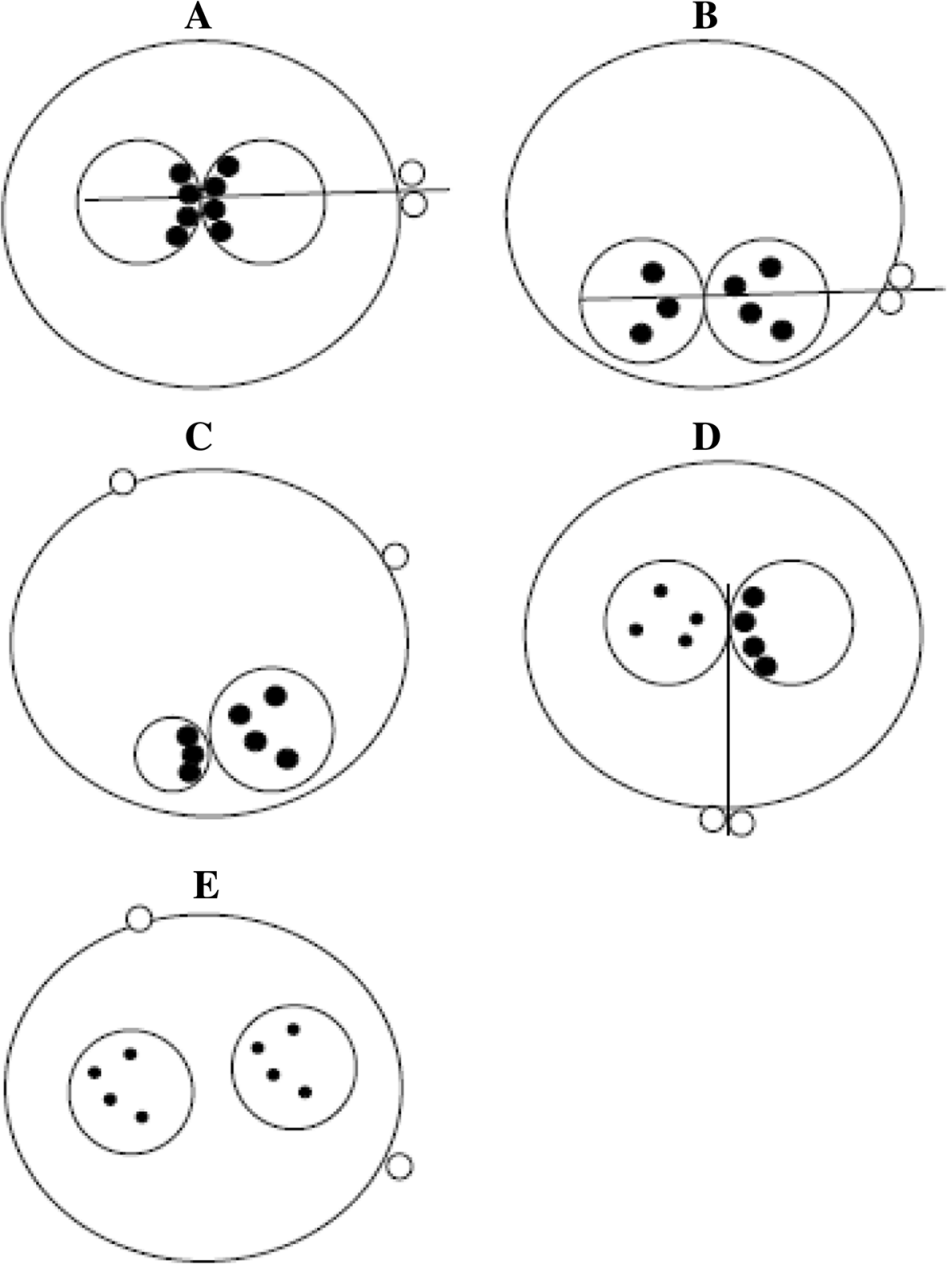
**Zygote scoring system of Nicoli et al. **[38]**.** Z1 group included zygotes with PN juxtaposed and centralized, nucleoli of large size, aligned and with the polar bodies aligned and oriented in the longitudinal axis **(A)**; Z2 group includied zygotes showing PN juxtaposed and peripheral, nucleoli of large size scattered in both PN, and polar bodies orientated in the longitudinal axis of the PN **(B)**; and Z3 group included zygotes show a different PN morphologies (centralized and separated, of unequal size and fragmented), different position of nucleoli (large size and scattered in both PN, large size and aligned in one PN and scattered in the other, and small sized in at least one PN and scattered) and different polar bodies orientation (perpendicular to the longitudinal axis; in different positions) **(C**-**E)**.

In 2007, Nicoli et al. [38], according to Scott’s [10] and Gianaroli’s [19] scores, developed a new scoring system. Zygote were classified into three Z-score groups: Z1 group including zygotes with PN juxtaposed and centralized, NPBs of large size, aligned and with the PBs aligned and oriented in the longitudinal axis (Figure 16A); Z2 group including zygotes showing PN juxtaposed and peripheral, NPBs of large size scattered in both PN, and PBs orientated in the longitudinal axis of the PN (Figure 16B); and Z3 group including zygotes show a different PN morphologies (centralized and separated, of unequal size and fragmented), different position of NPBs (large size and scattered in both PN, large size and aligned in one PN and scattered in the other, and small sized in at least one PN and scattered) and different PBs orientation (perpendicular to the longitudinal axis; in different positions) (Figure 16C-E) [38].

#### ESHRE scoring system

Zygote morphology is summarized in three categories: symmetrical, non-symmetrical and abnormal.

**Figure 17:**
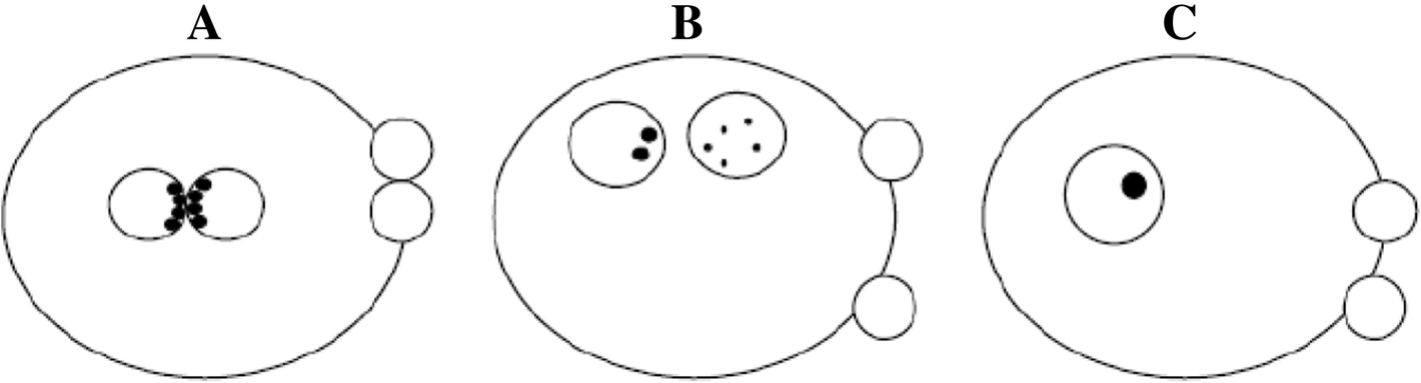
**ESHRE zygote scoring system **[1]**.** symmetrical zygotes show two PBs, two centrally located and juxtaposed PNs with distinct membranes, equal size and equivalent numbers and size of NPBs equatorially aligned at the region of membrane juxtaposition **(A)**, non-symmetrical zygotes (differing from the ideal configuration A) **(B)**, and abnormal zygotes with no NPBs and those with a single NPB **(C)**.

The symmetrical category include all zygotes showing two PBs, two centrally located and juxtaposed PN with distinct membranes, equal size and equivalent numbers and size of NPBs equatorially aligned at the region of membrane juxtaposition (Figure 17A). All the zygotes differing from this ideal configuration are included in the non-symmetrical category (Figure 17B). Finally, the abnormal category include zygotes with no NPBs and those with a single NPB (Figure 17C) [1].

### Zygote morphology and outcome measures

In Table 1 are summarized the results of the papers included in the final analysis, whereas in Table 2 details the risks for each endpoint to be or not to be related with zygote morphology.

**Table 2:** Risk to have or not to have a correlation between zygote morphology and ART outcomes

**Outcome**	**Correlation (studies; n., %)**	**No correlation (studies; n., %)**	**OR (95% CI)**	***P***
Embryo quality	15/25 (60)	10/25 (40%)	2.25 (0.73 to 6.98)	0.160
Cleavage stage	15/20 (75%)	5/20 (26.3%)	9.0 (2.15 to 37.66)	0.003
Blastocyst stage	7/8 (87.5%)	1/8 (12.5%)	49.0 (2.53 to 948.67)	0.010
Implantation rate	12/23 (52.2%)	11/23 (47.8%)	1.19 (0.37 to 3.79)	0.768
Pregnancy rate	12/25 (48%)	13/25 (52%)	0.85 (0.28 to 2.58)	0.777
Delivery/Live birth rate	1/4 (25%)	3/4 (75%)	0.11 (0.005 to 2.73)	0.179
Embryonic chromosome status	6/6 (100%)	0/6 (0%)	169.0 (2.89 to 9876.12	0.013

*OR*: odds ratio, *CI*: confidence interval.

In 38 [10-12,14-24,26-49] out of 40 (95.0%) studies included in the final analysis, zygote morphology was a part of cumulative morphological score during embryo development, whereas in the other 2 studies [13,25] (5.0%) zygote morphology was the only parameter for embryo transfer.

Table 3 shows the correlation between the ARTs outcomes and the time of the published studies.

**Table 3:** **Spearman’s rank correlations (*r; P*) between the publication year of the papers and the clinical efficacy of zygote morphology assessment**

**Outcome**	**Spearman’s correlation (*r*)**	***P***
Embryo quality	0.712	0.280
Cleavage stage	0.546	0.340
Blastocyst stage	0.659	0.417
Implantation rate	0.378	0.543
Pregnancy rate	0.129	0.047
Delivery/live birth rate	0.713	0.434
Embryonic chromosome status	0.815	0.923

### Biological outcomes

#### Embryo quality

Twenty-five studies out of 40 (62.5%) analysed the correlation between the zygote morphology and the embryo quality. Of these, 15 (60.0%) found a correlation [10,11,13,14,19,21,23,26],[27,29,33,34,37,39,40], while 10 studies (40.0%) did not find any correlation [12,17,25,28,30,38,39,41],[43,46].

Scott et al. [10], analysing a total of 3,701 zygotes, showed that the transfer of embryos deriving from Z1 and Z2 zygotes significantly increase the implantation and clinical pregnancy rates (see below) suggesting a significant efficacy on embryo selection [10]. The same zygote scoring system was subsequently used by Liu et al. [39] on 2,836 zygotes. In accordance with previous study [10], the Authors showed more excellent quality in embryo derived from Z1 and Z2 zygotes [39].

Gianaroli et al. [19,33] also found a significant correlation between the zygote morphology and embryo quality. On the contrary, data reported by Salumets et al. [15], after the analysis of a total of 2,284 zygotes, did not suggest any significant relationship. In agreement with Salumtes et al. [15], James et al. in 2006 [30] and Nicoli et al. in 2010 [46] found a lack of predictive value of zygote morphology on embryo quality. The former study [30] evaluated 3,333 zygotes with the scoring system described by Scott in 2003 [10], while the latter [46] evaluated 1,078 zygotes with the scoring system described by Gianaroli in 2003 [19].

#### Cleavage stage

Twenty studies out of 40 (50.0%) investigated the correlation between the zygote morphology and the embryo cleavage stage. Of these, in 15 (75.0%) studies was found a correlation [14,15,18,19,21-24,26,27,29],[32,40-42], whereas in 5 studies (25.0%) it was not found [12,17,28,47,48].

In 2001, Salumtes et al. [15] showed a statistically significant correlation between the zygote morphology and the embryo cleavage with the use of the scoring system proposed by Scott and Smith [50]. In 2003, Gianaroli et al. [19] concluded that the development of good-quality embryos was effectively dependent on the pattern of PN. In the same year, Scott et al. [22] and Lan et al. [23] published other two retrospective studies in which the same scoring system [10] was used and zygotes were scores at the same time (hours post-insemination). In the first paper, the analysis of 3,882 zygotes showed that the pattern of NPBs had a direct effect on embryo development [22]. In the second one, after the study of 1,894 zygotes, the Authors concluded that zygote score allows to select the most competent embryos to transfer [23]. More recently, Zamora et al. [44] confirmed the correlation between NPBs and the cleaved embryos on day 2 in a prospective study conducted on 2,105 zygotes [44].

Despite several studies seems to prove an effective correlation between zygote morphology and the embryo development, the issue is still open. In fact, in the two more recent papers by Nicoli et al. [46] in 2010 and Aydin et al. [48] in 2011, this association was not confirmed. In both papers the Authors concluded that the zygote morphology assessment have a limited significance in the choice of the best embryos to transfer [46,48].

#### Blastocyst stage

The correlation between zygote morphology and blastocyst stage was investigated in 8 articles out of 40 (20.0%). Seven of these (87.5%) reported a correlation [10,14,18,22,23,27,31], whereas only one (12.5%) did not [35].

The most recent study showing a significant correlation between the zygote morphology and the blastocyst stage was published by Sjöblom et al. [31] in 2006. In this study, the analysis of 1,961 zygotes showed a strong correlation with PN, NPBs features and the blastocyst development. Thus, the Authors concluded that the evaluation of zygote morphology can improve the embryo selection [31].

That data [31] confirmed findings previously reported by other Authors. In fact, Scott et al. [22] and Lan et al. [23] already described a significant correlation between the zygote morphology and the reaching of the blastocyst stage *in vitro*.

In spite of the above reported data, the most recent study by Guerif et al. [35] claimed that when combined with other embryo parameters, the zygote morphology did not correlate with blastocyst development. The Authors evaluated a very large sample of 4,042 zygotes with a modified from scoring system by Tesarik and Greco [51].

#### Embryonic chromosome status

All the studies (6/40, 15.0%) included in this systematic review and investigating the relation of the embryonic chromosome status and the zygote morphology found a close correlation [19,20,24,27,29,33].

In 2003, Gianaroli et al. [19] analysed 496 day 3 embryos by multicolour fluorescence in situ hybridization (FISH) to investigate the status of the chromosomes X, Y, 13, 15, 16, 18, 21 and 22 in patients submitted to pre-implantation genetic diagnosis (PGD). The Authors clearly showed that euploid status were only detected in embryos developed from good quality zygotes [19]. Similarly, Chen et al. [20] analysed with FISH 98 embryos to study their status of chromosomes X, Y and 18, concluding that the zygotes classified as Z1 led to a higher proportion of normal diploid embryos [20].

Data reported by Gianaroli et al. [19] and Chen et al. [48], were partially supported by Gámiz et al. [24]. In this paper, 569 day 3 embryos with ≥5 nucleated blastomeres and a ≤25% of fragmentation were submitted to FISH to study chromosomes X, Y, 13, 21, 16, 22 and 18 [24]. A significant correlation with zygote morphology was described, even if only in patients ≤37 year old [24].

In 2007, Gianaroli et al. [33] analysed the chromosomal status of day 3 embryos by the analysis of chromosomes X, Y, 13, 15, 16, 18, 21 and 22 performed by FISH, and confirmed their previously reported data [19] suggesting that zygote morphology was related to embryonic euploidy.

### Clinical outcomes

#### Implantation rate

Twenty-three studies out of 40 (57.5%) have tried to elucidate the possible prognostic value of the zygote morphology in the prediction of implantation. Twelve (52.2%) found a correlation [10,12,14,16,19,21,23,33],[36,39,40,43] between zygote morphology and implantation rate, while 11 studies (47.8%) did not find any correlation [15,17,26,28,30,32,34,38],[42,45,47].

Two studies published by Gianaroli et al. [19,33] reported a significant efficacy of zygote morphology to predict embryo implantation. The first was conducted in 2003 on 631 zygotes and the second one in 2007 on 4,042 zygotes. Two studies published more recently by Scott et al. [36] in 2007 and Liu et al. [40] in 2008 confirmed Gianaroli’s results. Grading the zygotes with the system proposed by Scott [10], both studies found a significant correlation between zygote morphology and implantation rate.

On the other hand, several other studies have been published showing no relationship between zygote morphology and implantation rate. Chen et al. [32] in 2006 showed that among the 1,186 zygotes evaluated, the best quality ones achieved the highest implantation rates, but the association did not achieve the statistical significance. In 2007 and in 2010, Nicoli et al. [38] (including 1,032 zygotes) and Weitzman et al. [45] (including 852 zygotes), respectively, confirmed the lack of statistically significant correlation between zygote morphology and rate of implantation. Finally, the most recent study conducted by Bar-Yoseph et al. [47] in 2011 on 1,516 zygotes, concluded that zygote scoring was not a good predictor of implantation.

#### Pregnancy rate

The correlation between zygote morphology and pregnancy rates has been investigated in 25 articles out of 40 (62.5%). In 12 cases (48.0%) that correlation was found [10-14,16,19,21,23,36,39,43], while in the other 13 (52.0%) any correlation was observed [15,17,25,26,28,30,32,34],[38,40,42,46,49].

The clinical efficacy of zygote morphology in the pregnancy prediction is one of the most studied and controversial issue. From 2000 to 2003, the majority of the studies published showed that the zygote morphology significantly correlated with the pregnancy rates [10-14,19,21,23]. In the subsequent years until today, this trend has significantly changed, due to the publication of studies in contrast with earlier data [25,26,28,30,32,34,38,40],[46,49].

Pregnancy rate was significantly influenced by publication year of the papers (*r*=0.129; *P*=0.047) (Table 3).

#### Delivery and/or live-birth rate

Four studies out of 40 (10.0%) aimed to elucidate the possible prognostic value of the zygote morphology in the prediction of delivery and live-birth rates. One study (25.0%) found a significant correlation [36], whereas 3 (75.0%) did not find any relationship [30,46,49].

The only study finding a significant correlation between zygote morphology and delivery/live-birth rates was published by Scott et al. [36]. The study was prospectively conducted on a total number of 2,528 zygotes with the scoring system proposed by the same Authors [10]. The Authors concluded that zygote morphology has a significant impact both on delivery and live birth rates in ARTs procedures [36].

Other three studies [30,46,49] investigating the correlation between the zygote morphology and delivery rate were retrospectively conducted and any significant correlation was found. Specifically, James et al. [30] in 2006 analyzed 3,333 zygotes claiming that embryos with different zygote features have similar viability, and so the zygote morphology evaluation does not affect the IVF/ICSI outcomes [30]. The lack of clinical significance of zygote morphology in the prediction of delivery and live-birth rate was confirmed by the two more recent analyses, published by Nicoli et al. [46,49] in 2010 and in 2013. The Authors included 1,078 zygotes in the first study and 755 zygotes in the second one [46,49]. Of note, this last study [49] was performed on 755 non-elective transfers of only one embryo, allowing a direct correlation between the zygote morphology and ARTs outcomes [49].

## Discussion

To our knowledge, the current is the first systematic review aimed to assess the effectiveness of the zygote morphology evaluation in fresh IVF and/or ICSI cycles.

Overall, the analysis of available data, obtained from 40 papers, showed a significant correlation between zygote morphology, cleavage stage and blastocyst stage. Moreover, albeit the zygote morphology was related with biological outcomes, the scenario resulted different at the regard of the clinical outcomes. In fact, clinical data about the relationship between zygote morphology and rates of implantation, pregnancy, delivery/live-birth were conflicting. Thus, to date it is not possible to draw conclusive answers on the usefulness of zygote morphology as tool for predicting clinical outcomes in infertile patients whom underwent to IVF/ICSI programs.

To the regard of the relationship between zygote morphology assessment and the embryonic euploidy status, available data did not permit to reach definitive conclusions too. In fact, despite all included papers showed a good predictive value of zygote morphology for embryonic chromosomal status, that results were obtained from analysis of only few and heterogeneous studies. In fact, only 6 out of the 40 included studies evaluated the correlation between zygote morphology and embryonic chromosomal abnormalities, and several biases were present, i.e. patients selected for PGD and/or maternal age, number of blastomeres, type and number of chromosomes analyzed [19,24,33].

Current data on the usefulness of zygote morphology in IVF/ICSI procedures in which embryos are transferred from day 2 to day 6 reveled that zygote assessment gives limited additional information for the selection of the most competent embryos to transfer [5,35,45].

Interestingly, the analytic analysis of available data suggested an influence of the publication year on the studies’ results. Specifically, a significant effect of the publication year was detected for the relationship between zygote morphology and rates of pregnancy, whereas this effect did not achieve the statistical significance for other end-points assessed. This figure could be explained with a possible initial over-estimation of the efficacy of the zygote morphology scoring in the beginning of its application in the clinical setting.

The main limitation of this systematic review was the inclusion of studies using different methods of zygote morphology classification [5,10,50,51]. In fact, our search identified many zygote morphology scoring systems. This bias made not possible not only a data synthesis but also any comparisons among results from different studies. To this regard, in 2011 there was a consensus conference of the Alpha Scientists in Reproductive Medicine and ESHRE Special Interest Group of Embryology aimed to standardize the zygote morphology assessment, and three zygote categories were defined [1].

In no study included in the current review was assessed the reproducibility of any of the zygote scoring system and no formal validation study in other settings was available in literature. Moreover, there are not studies conducted to verify the efficacy and the reproducibility of the more recent classification proposed by the ESHRE [1]. In addition, only in few studies a single embryo transfer (SET) was performed. Thus, it was not possible to define formally a close and direct link between zygotes morphology, implanted embryos and all other clinical IVF/ICSI outcomes. Finally, only few available data [13,25] were available on the use of the zygote morphology as the only parameter for embryo selection.

Other two important points need to be discussed, i.e. the time of zygote observation and the insemination procedure. This review showed that the time of zygote check was extremely variable within and among the different studies ranging from 12 hours [21] to 23 hours after insemination [34]. Moreover, data reported in the literature clearly suggested that the timing of zygote observation can not be the same for zygotes derived from IVF and from ICSI. In fact, since spermatozoa used for IVF have been pre-incubated during the capacitation process, zygotes arising from IVF should be observed one hour behind those arising from ICSI [1,2,53]. So, a standardization in the observation timing is necessary to compare data from different studies. To this regard, the actual recommendation is to check the zygote morphology 17±1 hours after the insemination, taking into account the insemination procedure [1,3].

Another point of discussion is the high dynamicity of the pronuclear formation [9]. In fact, due to the dynamic nature of processes bringing to PN formation and development, the zygote morphology is hardly resalable in a single static evaluation [5,48]. To overcome the limitations derived from the single and static observation with invertoscope, the time-lapse imaging system (TIS) has been recently suggested [5]. This technology is increasingly used in the laboratory to select the most competent embryos not only on the basis of morphological features, but also observing the kinetic of embryo development. This specific approach is known as morphokinetic analysis [54].

An increasing number of publications reports on the variation of the time points for specific developmental stages as prognostic markers of embryo competence [5,55]. Moreover, the application of TIS and morphokinetic analysis has been also recently described in order to identify the risk of aneuploidy embryos [56,57]. Thus, the TIS use could define not only the best timing for zygote assessment but also definitively clarify if, how and how much the zygote morphology and dynamic changes influence the success of IVF and/or ICSI procedures.

In conclusion, current systemic review failed to achieve conclusive results on the usefulness of the assessment of zygote morphology in ARTs. However, zygote check is the only one way to verify the presence of abnormal fertilization (1 PN or more then 2 PN) [1,2] and zygote morphology evaluation is a useful tool to selected the best zygote to transfer and/or freezing at day 1 [18], even if no evidence-based suggestion can be given on the best scoring system to use in the clinical practice.

## Abbreviations

ARTs: Assisted reproductive technologies; CI: Confidence interval; FISH: Fluorescence *in situ* hybridization; ICSI: Intracytoplasmic sperm injection; IVF: In-vitro fertilization; NPBs: Nucleolar precursor bodies; OR: Odds ratio; PBs: Polar bodies; PGD: Preimplantation genetic diagnosis; PN: Pronuclear.

## Competing interests

The authors report no conflict of interest and any source of financial support for the research.

## Authors’ contributions

AN conceived of the study and helped to draft the manuscript; SP conceived of the study, and participated in its design and coordination, and wrote and revised the manuscript; FC participated in data collection and analysis, wrote and revised the manuscript, MF participated in data collection and analysis, AF performed the statistical analysis and helped to draft the manuscript; GBLS conceived of the study and helped to draft the manuscript. All authors read and approved the final manuscript.
